# Quantitative Elasticity of Flexible Polymer Chains Using Interferometer-Based AFM

**DOI:** 10.3390/nano12030526

**Published:** 2022-02-03

**Authors:** Vikhyaat Ahlawat, Surya Pratap S. Deopa, Shivprasad Patil

**Affiliations:** Department of Physics, Indian Institute of Science Education and Research (IISER) Pune, Pashan Road, Pune 411008, India; vikhyaat.ahlawat@students.iiserpune.ac.in (V.A.); suryapratap.deopa@students.iiserpune.ac.in (S.P.S.D.)

**Keywords:** AFM, oscillatory response, persistence length

## Abstract

We estimate the elasticity of single polymer chains using atomic force microscope (AFM)-based oscillatory experiments. An accurate estimate of elasticity using AFM is limited by assumptions in describing the dynamics of an oscillating cantilever. Here, we use a home-built fiber-interferometry-based detection system that allows a simple and universal point-mass description of cantilever oscillations. By oscillating the cantilever base and detecting changes in cantilever oscillations with an interferometer, we extracted stiffness versus extension profiles for polymers. For polyethylene glycol (PEG) in a good solvent, stiffness–extension data showed significant deviation from conventional force–extension curves (FECs) measured in constant velocity pulling experiments. Furthermore, modeling stiffness data with an entropic worm-like chain (WLC) model yielded a persistence length of (0.5 ± 0.2 nm) compared to anomaly low value (0.12 nm ± 0.01) in conventional pulling experiments. This value also matched well with equilibrium measurements performed using magnetic tweezers. In contrast, polystyrene (PS) in a poor solvent, like water, showed no deviation between the two experiments. However, the stiffness profile for PS in good solvent (8M Urea) showed significant deviation from conventional force–extension curves. We obtained a persistence length of (0.8 ± 0.2 nm) compared to (0.22 nm ± 0.01) in pulling experiments. Our unambiguous measurements using interferometer yield physically acceptable values of persistence length. It validates the WLC model in good solvents but suggests caution for its use in poor solvents.

## 1. Introduction

Single-molecule force spectroscopy (SMFS) experiments are indispensable in studying biomolecules and other polymeric complexes at the single-molecule level [1]. The manipulation of single molecules with high force sensitivity and spatial resolution allows understanding of intramolecular and intermolecular interactions of proteins and polymers. In an SMFS experiment, the force–extension curve (FEC) probes the conformational landscape of molecules along a well-defined reaction coordinate. Both the thermodynamic free energy of the landscape [2] and conformational dynamics over the landscape [3,4] can be extracted using FEC.

However, single-molecule force–extension curves are sensitive to artifacts and can be misinterpreted as a valid single-molecule trajectory. In the recent past, a number of experiments [5,6,7,8,9] and simulations [10,11] have considered separating intrinsic thermodynamic and kinetic signatures of molecule from effects of instrument. These studies consider effects like finite response time of AFM cantilever probe and/or its stiffness on the accuracy of extracting parameters of a molecule’s landscape.

A common way of generating FEC using AFM is to perform pulling experiments in constant velocity mode. In this, one end of polymer is tethered to the cantilever tip while another end of the polymer is held by the substrate. The cantilever is then displaced at a constant velocity with respect to the substrate to generate FEC. In good solvent conditions, the relationship between force and extension of polymer is linear but beyond a certain force it becomes non-linear. Typically, this non-linear force–extension region is measured in AFM experiments and modeled with two classes of models i.e., the worm-like chain (WLC) and freely jointed chain (FJC) model. The WLC model considers the chain as a continuous string and parameterizes the chain stiffness with persistence length $l_{p}$, characterizing the chain’s local flexibility. Persistence length is usually estimated from fitting the WLC model to the experimental force–extension data. Importantly, the value of persistence length using constant velocity pulling experiments reported in the literature is anomaly low [12,13,14,15,16] and is sometimes smaller than the size of a single monomer. FJC and its variations are used to justify low values of persistence length [12,16,17]. FJC considers that the polymer chain is made up of rigid segments characterized by the Kuhn length $b=2l_{p}$. These segments are completely uncorrelated with each other [18,19] and therefore this model is unphysical compared to the exponential decay of correlation between tangent vectors along a WLC chain.

Instead of global pulling experiments, a local oscillatory protocol is better suited for the sampling of the conformational landscape. This is due to two reasons: (1) the oscillatory technique is bidirectional in nature and hence is accurate in sampling the conformational space [20], (2) extracting an oscillatory response allows for a simple interpretation of intrinsic elasticity from the convolution of instrument effects [21]. However, oscillating the cantilever base end with a piezo and using the optical beam deflection scheme to detect oscillations necessitates viewing the cantilever as a continuum beam. This is primarily because the beam deflection scheme detects changes in the slope of the cantilever at its tip-end. A beam theory approach based on a fourth-order partial differential equation is typically used to describe the cantilever oscillating hydrodynamics under Euler–Bernoulli assumptions. This includes assumptions regarding boundary conditions and geometrical shape of beam [22]. In practice, these assumptions may not be satisfied and can lead to misinterpretation. In addition, it is difficult to precisely account for hydrodynamics of cantilever while operating in liquid environment [22]. This can lead to artifacts [23]. Here, we use a home-built fiber-based interferometer detection scheme to avoid the complexity of interpretation. Due to local and direct detection of cantilever displacement rather than slope change, it allows the use of a simple point-mass description of cantilever dynamics. The equation of motion for dynamics of a point mass is the classical damped simple harmonic oscillator (SHO). Hence, a straightforward and universal description based on SHO is suitable for an accurate estimate of elastic response.

In this work, we use a fiber-interferometer-based AFM to measure the elastic response of flexible polymers. Measurements were made on PEG and polystyrene in good and poor solvents. Along with pulling on the polymer with relatively low constant velocity, sub-nanometer oscillatory perturbations were applied to extract the elastic response. The measured response was interpreted with the WLC model of entropic elasticity and persistence length was extracted. It shows significant deviation from conventional pulling experiments for PEG (0.5 ± 0.1 nm) and polystyrene in good solvents (0.88 ± 0.02 nm) but no deviation is observed for polystyrene in a poor solvent. In addition, the fluctuations about a mean elastic response showed a large variance for a shorter-length polymer chain. The results were rationalized with statistical mechanics of the combined cantilever–polymer system.

## 2. Materials and Methods

### 2.1. Sample Preparation

Polystyrene of molecular weight 192 KDa was purchased from Merk (Sigma-Aldrich Chemicals Private Limited, Bangalore, India) and dissolved in THF (tetrahydrofuran) to μM concentration. Thereafter, a drop of 60 μL was incubated on a clean glass coverslip and later cleaned excessively with THF solvent. After drying the coverslip, it was loaded into the fluid cell and filled with water and 8M Urea for experiments in respective solvents. For experiments with PEG, a 10 KDa molecular weight was purchased in powder form and dissolved in Milli-Q water (18 MΩ cm) to 1 mM concentration. A sample of about 80 μL was then incubated for half an hour on a freshly prepared gold coverslip. The coverslips were prepared from thermal evaporation deposition and first treated with UV ozone to remove organic impurities before use. The thiol terminated PEG is then able to form a covalent bond with a gold surface. This procedure not only results in strong attachment but also gives off a large rupture force when the polymer detaches from the AFM tip. The sample was rinsed clean with Milli-Q before mounting it in a fluid cell for further measurements in water.

### 2.2. Fiber-Interferometer AFM

In fiber-interferometer-based AFM [24,25,26], three major assemblies work in conjunction to determine its overall operations.

**Figure 1:**
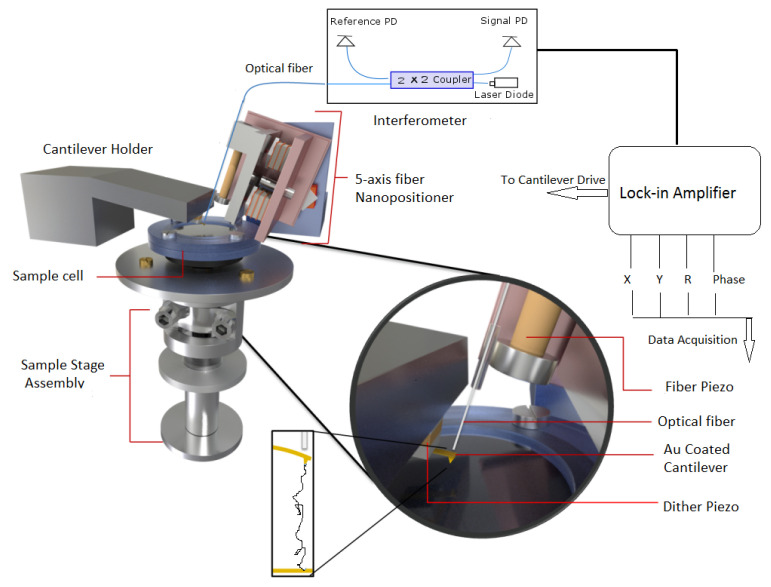
A schematic diagram of AFM used in experiment. It consists of three major components (1) fiber-interferomer, (2) 5-axis nanopositioner, and (3) sample-stage assembly. A lock-in amplifier is used to detect amplitude and phase changes from signal photodiode (PD) output.

(i) Fiber-optics-based interferometer detector: This detector uses a single-mode fiber to detect an interference pattern formed from the combination of light reflected at the fiber end and cantilever end which are placed very close to each other. The interference pattern is very sensitive to separation between the cantilever and fiber and points of maximum sensitivity are chosen for the operation. To form the interference pattern, the cantilever surface and fiber end are made parallel to each other which is ensured by aligning the fiber perpendicular to the backside of the cantilever.

(ii) The second assembly is a 5-axis fiber slider nanopositioner [25], which is used for this precise alignment. As shown in Figure 1, it consists of two mutually perpendicular slider plates (shown in light red) each capable of moving in its plane and rotating about an axis perpendicular to the plane. The plates are driven by a stack of shear piezos, in sets of three, glued onto them and are connected to each other by magnetic screws for optimal sliding force. The yzϕ slider has polished sapphire plated glued to it, which slides against the sapphire balls attached on top of piezo-stacks for xzθ slider. By providing logical voltage pulses to piezo stacks, inertial sliding motion is initiated and sliders move in xzθ and yzϕ directions giving rise to motion along five independent axes (x,y,x,θ,ϕ). The optical fiber is attached to a steel plate holder which also holds a tube piezo (yellow part in Figure 1) for vertical motion of fiber. This plated holder is moved by piezostacks of yzϕ slider as depicted in Figure 1. Thus, the 5-axis slider can precisely position the fiber very close and perpendicular to the cantilever backside for a good interference pattern.

(iii) For force spectroscopy experiments, the sample stage assembly is required to be approached or retracted from the cantilever tip. As shown in Figure 1, it is done using scanner and hammer tube piezo for finer and coarser motion respectively. The sample holder is mounted on a scanner piezo tube which is enclosed in a glass tube. The end of this glass tube is attached to a hammer tube piezo and the glass tube itself is held in place by leaf spring. The bottom end of the hammer piezo tube supports a steel disk (hammer disk) and provides the necessary inertia for coarser motion along the vertical z directions. The hammer piezo when given large voltage pulses with slow rise and rapid fall give rise to coarser motion along the z direction. During the slow rising part, hammer piezo contracts but this does not disturb the glass tube attached at its top end and held by leaf spring. However, with sharp fall, the piezo suddenly expands and this is opposed by the inertia of hammer disk which forces glass tube to overcome leaf spring and slide against it. Similarly, the external electrode of the scanner piezo tube is segmented into four quadrants and the application of suitable pulses produces finer x,y,z motion. For more details about the instrument, see ref. [25].

For measurements, a gold-coated cantilever purchased from micromesh were used. The cantilever stiffness and resonance frequency were 0.8 N/m and 13 KHz and stiffness was calibrated using thermal fluctuation measurements at room temperature 23 °C [27]. The cantilever was mounted on a holder with stacks of dither piezo beneath it. The vantilever was oscillated at an off-resonance frequency of ∼500 Hz using an internal oscillator from lock-in. The fiber was accurately aligned on the back of the cantilever using a 5-axis nanopositioner. The interference pattern so produced was used to determine the point of maximum sensitivity and was locked at this position using a feedback loop for further operations. Thereafter, the sample was approached towards the cantilever-tip using cantilever oscillating amplitude as set-point value. Once approached, the sample was retracted at constant velocity of 80 nm/s and output of signal photodiode was fed as input to lock-in amplifier to record amplitude *R*, phase θ, X (=*R*sinθ), and Y (=*R*cosθ) output of lock-in amplifier. All experiments were carried out at room temperature 23 °C. The stiffness–extension curves measured for PEG in water were 25 and for polystyrene in water and 8M Urea were 26 and 14, respectively. The concentration of polymers stock solution was low so that mostly single binding events with the cantilever tip were detected. The stiffness–extension profiles which could be normalized by their apparent contour length (obtained by fitting to WLC) were finally chosen for analysis.

### 2.3. Modeling the Dynamics of Cantilever–Polymer System

**Figure 2:**
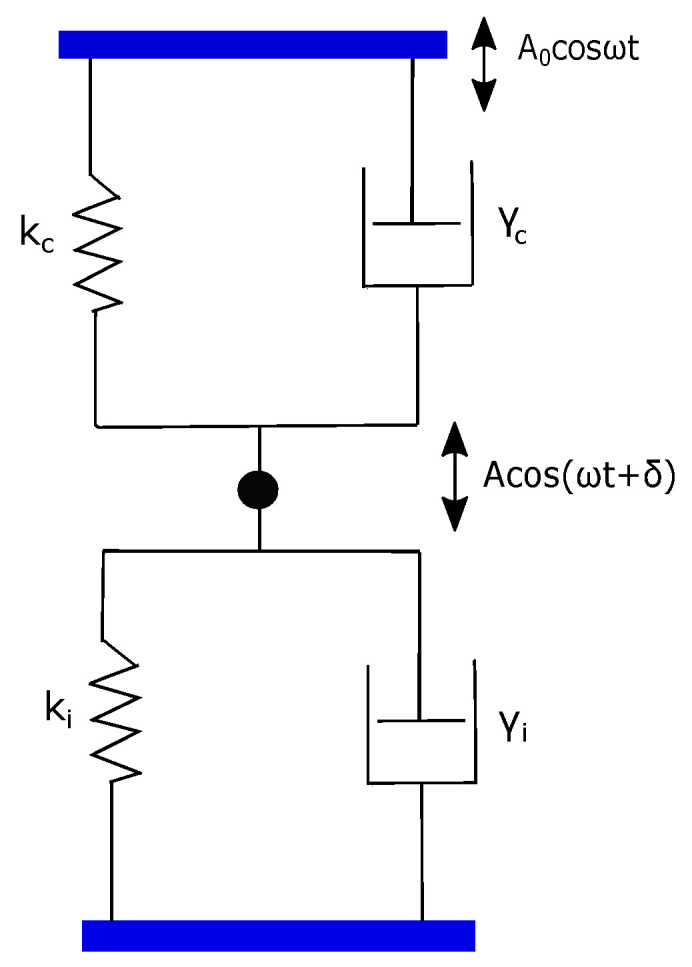
A model for cantilever plus polymer configuration. It consists of two Voigt elements ($k_{i}$,$\gamma_{i}$) and ($k_{c}$,$\gamma_{c}$) arranged in a parallel assembly.

For the fiber-interferometer method, a simple point-mass description becomes valid due to local detection at a point. Therefore, the dynamical response of the cantilever is well described by the damped simple harmonic oscillator (SHO) model. An SHO can be represented as a point mass *m* connected to a Voigt element consisting of spring $k_{c}$ and dashpot $\gamma_{c}$ of the cantilever. On the other hand, the response of polymer can be represented by a Voigt element with spring $k_{i}$ and dashpot $\gamma_{i}$. The model for overall cantilever–polymer configuration is two Voigt elements connected as shown in Figure 2 [28,29].

In this parallel assembly, the cantilever and polymer contribution simply add up to an effective $k=k_{i}+k_{c}$ and an effective $\gamma =\gamma_{i}+\gamma_{c}$. This is justified on the basis that the polymer and cantilever have equal extension Δx but they both experience independent forces. Therefore, the net force due to both cantilever and polymer springs, for instance, is −($k_{i}+k_{c}$)Δx where Δx is cantilever or polymer extension.

Accordingly, the combined dynamics of cantilever plus polymer due to base dithering is described by:

(1) $$m^{*}\ddot{z}+\gamma \dot{z}+kz=A_{0}k_{c}\cos\omega t$$

where $A_{0}$ is amplitude of cantilever dithering in absence of polymer. For off-resonance frequency, $\omega <<\omega_{0}$, where $\omega_{0}^{2}=k_{c}/m^{*}$, it can be shown that dissipative $\gamma \dot{z}$ and inertial term $m^{*}\ddot{z}$ are negligible compared to kz term [30]. Under the assumption $k_{c}>>k_{i}$, a steady state solution z=Acosωt+δ, give:

(2) $$A=A_{0}\left(1-\frac{k_{i}}{k_{c}}\right)\;\mathrm{and}\;\delta ∼0$$

Here *A* is the cantilever amplitude and δ is the phase difference between cantilever drive and cantilever oscillations. The quantity $\gamma \dot{z}$ does not dominate in off-resonance conditions and hence we expect zero phase difference. This condition, therefore, forms a check on the correctness of our measurement. We have recently shown that for off-resonance operation, phase difference δ in the displacement signal *z* is 0 [23,31]. The amplitude signal gives the stiffness of the polymer molecule and both amplitude and phase are measured with a lock-in amplifier.

## 3. Results and Discussion

### 3.1. Polyethylene Glycol (PEG)

As a polymer is pulled with AFM, force-extension curves (FECs) report the entropic nature of polymer elasticity due to vast changes in conformational space. These curves are usually modeled with two main classes of entropic models i.e., the worm-like chain (WLC) and (FJC) freel-jointed chain models. In the WLC model, the polymer chain is considered to be a continuum string and a parameter called persistence length ($l_{p}$), characterizes chain local flexibility. At low force stretching, the relation between force and extension is linear (*x*∼*f*) and progressively becomes non-linear ($1-\frac{x}{L}∼\sqrt{\frac{k_{B}T}{Fl_{p}}}$) as extension approaches polymer contour length *L*. An exact analytical expression between force and extension is not possible for WLC. However, a Marko–Siggia interpolation formula [32] describes the WLC behavior accurately in all force regimes as;

(3) $$F=\frac{k_{B}T}{l_{p}}(\frac{1}{4{\left(1-\frac{x}{L}\right)}^{2}}-\frac{x}{L}+\frac{1}{4})$$

**Figure 3:**
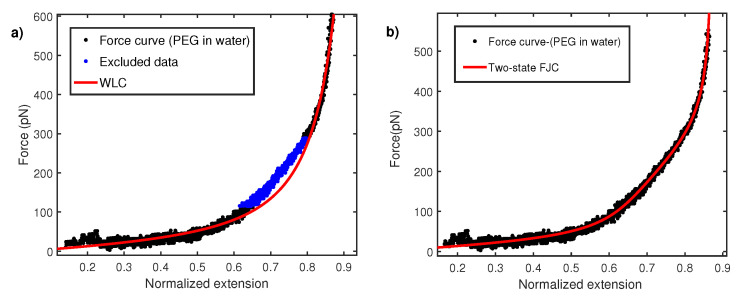
(**a**) Force–extension curve for PEG in water fitted with WLC with a persistence length $l_{p}=0.12\pm 0.02$ nm. A region between 100 and 300 pN does not fit and is also excluded. (**b**) Force–extension curve fitted with two-state FJC model with kuhn segment length 0.24±0.02 nm.

Here, *x* is the extension of molecule in nm measured via calibrated displacement of the AFM sample base with respect to the AFM cantilever. The persistence length is estimated from fitting above WLC in relation to experimental measured FEC. It turns out that a wide variety of polymers show anomaly low value of persistence length in AFM constant velocity pulling measurements [12,13,14,15,16]. The persistence lengths are even smaller than the size of monomer units. Figure 3a shows the normalized force–extension profile for polyethylene glycol (PEG) taken in water (Milli-Q). The normalization procedure is carried out by fitting WLC to experimental data and extracting the apparent contour length in each fit. The overall extension of the molecule is normalized with this contour length. When modeling FEC with WLC, it is observed that there is a region between 100 and 300 pN where WLC does not fit well. As explained later, the reason for this behavior is the conformation transition of PEG monomers in water. However, the overall fitting shown in Figure 3 give a persistence length of 0.12 ± 0.02 nm. This persistence length is even smaller than the c-c bond length (0.16 nm) and therefore is physically unrealistic. In order to explain this, a two-state model based on FJC has been put forward. A PEG monomer in water undergoes length change from a shorter gauche state to a larger all-trans state under strong stretching force [33,34,35]. The two-state FJC model [33] incorporate FJC entropic elasticity with length change from gauche (shorter conformer) $L_{gauche}$ to trans (longer conformer) $L_{trans}$. These two states are separated by the free energy barrier of ΔG. In this model, relative extension *z* is given by:

(4) $$z=\left[\frac{L_{gauche}}{e^{\frac{-\Delta G}{k_{B}T}}+1}+\frac{L_{trans}}{e^{\frac{\Delta G}{k_{B}T}}+1}\right]*z_{fjc}/L_{trans}$$

$\mathrm{where}\;\Delta G=\left(G_{trans}-G_{gauche}\right)-F\left(L_{trans}-L_{gauche}\right)\;\mathrm{and}\;z_{fjc}=coth\left(\frac{Fb_{k}}{k_{B}T}\right)-\left(\frac{k_{B}T}{Fb_{k}}\right).$

Here $L_{trans}$ is fixed 0.256 nm, the length of two repeating C-C bond lengths. This equation has three free parametes, ΔG, $L_{gauche}$, and Kuhn length $b_{k}$. The effective increase in length reduces the stiffness of the polymer and therefore results in a nearly linear rather than a curved regime in the force–extension curve. Hence, two-state FJC is expected to provide a better fit to FEC data in intermediate force between 100 and 300 pN. Figure 3b shows fitting with the two-state model and yields a Kuhn length of 0.24 ± 0.02 nm or a persistence length of 0.12 nm. The persistence length of 0.12 nm is again lower than the c-c bond length and raises the question on the validity of fitting the data with the WLC model. The Kuhn length of 0.24 nm coincides with the value obtained in other polar solvents like 2-propanol [21] and others [36] but is about five times lower than its measurement with magnetic tweezers [37,38]. It is noted that an earlier work by Oesterhelt et al. [33] reported a Kuhn length of 0.7 nm in large size solvent molecule like hexadecane. This value was further used to fit the two-state FJC model for a PEG force–extension curve in water. It, however, turns out that a large size molecule can induce additional excluded volume effects, as shown in a recent study [36].

**Figure 4:**
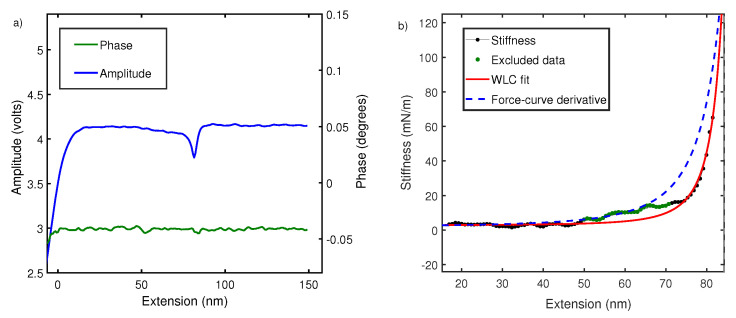
(**a**) The raw amplitude *A* and phase δ profiles measured using fiber-based interferometer for PEG in water. (**b**) Comparison of stiffness–extension curve measured from amplitude signal (black) with derivative of force–extension curve (blue dash). It also shows fitting of stiffness–extension curve with WLC (red) while excluding the region between 50 and 70 nm is shown in green. A similar behavior is seen in Figure 2 for force–extension curves. The persistence length estimated is 0.5±0.1 nm.

To understand this, we performed local oscillatory measurements on the polymer while it is pulled at a constant velocity ∼70 nm/s. The cantilever is oscillated by sinusoidal driving at the base with dither piezos. Cantilever amplitude and phase difference between cantilever drive and actual oscillations at the tip are measured using an interferometer and recorded usina g lock-in amplifier. As shown in Figure 4, phase difference is close to zero and featureless due to negligible contribution made by dissipation in off-resonance conditions [23,31,39]. However, amplitude signals show characteristic non-linear features every time a polymer is picked up. According to Equation (2), the amplitude signal is linearly proportional to the elastic response of polymer $k_{i}$ and the equation is therefore used to convert the amplitude–extension relation to the stiffness–extension relation. The stiffness–extension curve so generated (in black) is shown in Figure 4. As evident, there is a clear deviation between the stiffness–extension curve and the force–extension derivative (blue) obtained in global pulling experiments. WLC model is fitted to stiffness–extension data but the region between 50 and 70 nm does not fit well. This region corresponds to a linear region between 100 and 300 pN observed in force–extension curves and likely results from length transition for PEG monomer in water. The above result shows that our measurement is sensitive to a conformational change and yields a persistence length of 0.5 ± 0.1 nm.

This value of the persistence length (0.5 nm), which is obtained using oscillatory measurements, matches well with equilibrium magnetic tweezer measurements in low force regime [37,38,40]. Specifically, Innes-gold et al. [38] and Dittmore et al. [37] measured persistence lengths of 0.55 and 0.5 nm, respectively. In addition, ensemble measurements using neutron scattering and other bulk techniques [18,41] report a persistence length $l_{p}$ of 0.6 nm.

In the past, the viscoelasticity of single polymer chains has been determined by oscillatory response [42,43,44]. As opposed to the present work, these studies used oscillation frequencies close to the resonance of the cantilever and measured dissipation for a polymer from changes in phase lag. Recent efforts in correctly modeling the hydrodynamics of the cantilever suggest that single-polymer dissipation is likely a misinterpretation [23,45]. Specifically, it hints to a problem of distinguishing elastic response from that of dissipative for frequencies of oscillation close to the resonance of cantilever. Secondly, the deflection detection measures the slope of the cantilever which is prone to artifacts owing to the spurious phase lags produced due to a variety of reasons [23,45]. Oscillatory response of PEG was obtained previously in water [46]. Kienberger et al. functionalized the PEG chain end to attach it to the cantilever tip and employed a magnetic excitation method to oscillate the cantilever. The frequency of oscillation was chosen close to cantilever resonance and both pulling force–extension curve and stiffness–extension curve from oscillatory response yielded a similar persistence length of 0.38 nm. Compared to our measurement, this study is not strictly off-resonance, however, the magnetic excitation method is known to produce artifact-free measurements [47]. It is important to note that oscillatory measurements again produce a reasonable estimate of persistence length. A proper investigation is needed to compare Kienberger et al.’s method to the one used in the present work.

### 3.2. Polystyrene

Polystyrene, due to its homogeneous structure and hydrophobic side chain is proposed as an ideal homopolymer to study polypeptide (protein) hydrophobic collapse in physiological water [48]. A hydrophobic collapse is a first and critical step in the self-assembly of proteins, especially for globular proteins. A protein molecule tends to denature in high concentrations of aqueous urea and the dominant mechanism responsible for denaturation is the weakening of the hydrophobic region of proteins by urea. Therefore, urea is generally considered a good solvent for hydrophobic homopolymers and water a poor solvent [49,50,51]. In this part, we describe our local oscillatory measurement on polystyrene in water and 8M Urea.

**Figure 5:**
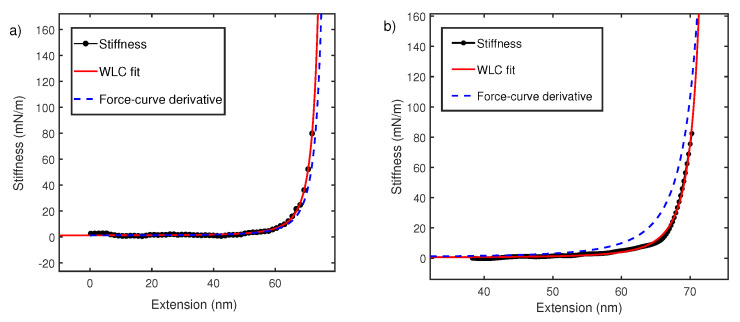
(**a**) Polystyrene stiffness–extension curve in water (black) measured using interferometer-based AFM and fitted with WLC model (red). It is compared with derivative of force–extension curve with $l_{p}$ 0.23 nm (blue dash). (**b**) Polystyrene stiffness–extension curve taken in 8M Urea (black) fitted with WLC (red) of $l_{p}=0.88\pm 0.02$ nm and compared with force–extension derivative (blue dash) of $l_{p}$ 0.23 nm.

A force–extension curve of polystyrene in constant velocity pulling experiments tends to show anomaly low values of persistence length. Polystyrene shows a persistence length ($l_{p}$) of 0.23 nm in the poor solvent of water [13] and 0.25 nm in a good solvent like toluene [16,52]. These values are not consistent with scattering experiments which expect a persistence length greater than 1 nm [18]. They are also at odds with the fact that there is a 0.72 nm long side group that can offer significant steric hindrances. Therefore, these values of persistence length are debated [16]. To address this, we measured the oscillatory response of polystyrene while it is pulled at a constant velocity. The results for polystyrene in water (Milli-Q) are depicted in Figure 5a. It shows that stiffness–extension curve (black) generated from amplitude signal of lock-in using Equation (2). This curve shows no noticeable deviation from the derivative of the force–extension curve (in blue dash) with $l_{p}$ 0.23 nm. The WLC fit (in red) to stiffness–extension data gives a persistence length of 0.26 ± 0.02 nm.

It is interesting to note that globular protein domains like I27 have been studied with both oscillatory and pulling experiments and similarly show no deviation [23,45,53]. The persistence length is also similar to that measured with magnetic tweezers [54]. In the next section, we explain this by noting an additional contribution from hydrophobic free energy, which makes fitting with WLC alone an ad hoc process. WLC only accounts for entropic elasticity of polymer backbone and no side chain effects are considered. In cases of poor solvents like water, persistence length is a heuristic parameter that may take a lower value to accommodate hydrophobic interaction and makes up for the inadequacy of models describing the polymer elasticity [55,56].

We next carried out similar experiments in 8M Urea. Figure 5b shows stiffness–extension curve (black) from oscillatory measurement which deviates significantly from derivative of force–extension curve (blue dash). When stiffness–extension data is fitted with the WLC model. This yields a persistence length of 0.88 ± 0.02 nm.

The data in Figure 5b, is consistent with our observation in Figure 4. It can be concluded that for polymer chain in good solvents, the stiffness measurement using oscillatory method deviates from the derivative of force–extension curves (Figure 4b and Figure 5b). The direct measurement of stiffness using oscillatory measurements yields reasonable values of persistence length, which are consistent with other techniques. We explain this observed deviation between constant velocity measurements and stiffness extension curves in the next section.

### 3.3. Explanation of Deviation

The polymer entropic elasticity in good solvent conditions is statistical in nature, arising from a distribution over various accessible conformations. In that sense, a proper interpretation of force–extension measurement requires a combined statistical mechanics of the polymer–cantilever system. The canonical partition function of the polymer molecule and cantilever are $Z_{m}$ and $Z_{c}$ respectively and they combine to give overall partition function $Z_{system}$ as [57,58,59]:

(5) $$Z_{system}∼Z_{m}\times Z_{c}=e^{-\beta F(x)}\times e^{-\beta k_{c}{\delta_{c}}^{2}/2}$$

Here β is 1/$k_{B}T$ and F(x) is free energy as a function of end-to-end length coordinate *x*. For cantilever harmonic biasing potential $k_{c}{\delta_{c}}^{2}/2$, the cantilever deflection $\delta_{c}$ is D−x where *D* is the displacement of the cantilever with sample surface at constant velocity and *x* is the end-to-end length of the polymer. Therefore, we see that $Z_{m}\times Z_{c}$ is a mathematical convolution which shows that both cantilever and polymer are acting simultaneously in an intricate convolution. This is likely to produce a biased trajectory for the polymer. One possibility to get an isolated response from $Z_{m}$ only is at high deflection or force where $Z_{c}$ reduces to a delta function δ(D−x). Another way to achieve this is to perform measurement with a constant force magnetic tweezer setup. These are effectively zero stiffness measurements in which case it can be shown that effects of cantilever integrate out to get an isolated response from $Z_{m}$ only [58,59]. This explains our match of persistence length with magnetic tweezer measurements and suggests that oscillatory measurement is sampling an intrinsic trajectory of polymer. The likely reason for intrinsic measurement is the parallel coupling pathway implemented by local oscillating measurements. For the total amplitude response *A* in Equation (2), the stiffness of cantilever $k_{c}$ and stiffness of polymer $k_{i}$ effectively add up in what is called parallel combination. This allows for a much clearer separation of cantilever contribution from polymer compared to intricate convolution.

For polystyrene in the poor solvent of water, we observed no deviation between force–extension and stiffness–extension curves. The reason for this is the large and positive hydrophobic free energy required to stretch a hydrophobic polymer in water [60]. This is in addition to the conformational entropy of the polymer backbone described by WLC in a good solvent. A polymer chain tends to minimize its accessible surface area and an extra hydrophobic free energy, which is at least six times the chain entropy, is required for stretching [55,56]. In absence of hydrophobic interactions, only WLC entropic contribution $F_{WLC}=-TS_{WLC}$ of about ∼2$k_{B}T$ determine free energy of polymer *F*. Additionally, the harmonic biasing potential is ∼7$k_{B}T$ for an intermediate force of 200 pN. This means that in Equation (4), the cantilever and polymer are almost equally weighted to the overall partition function $Z_{system}$ and likely produce a bias. However, a larger contribution from hydrophobic free energy, say at least $F_{hydro}$∼$20k_{B}T$ [61], due to bulky aromatic side group makes the weight of cantilever biasing nominal compared to free energy *F*. Hence, a more intrinsic response is sampled with no deviation from the stiffness–extension curve of oscillatory measurement. Importantly, WLC fitting only produces persistence length as an effective parameter to account for the inadequacy of the WLC model in a poor solvent. This results in a lower value of persistence length and perceived softening of polymer [55,56].

**Figure 6:**
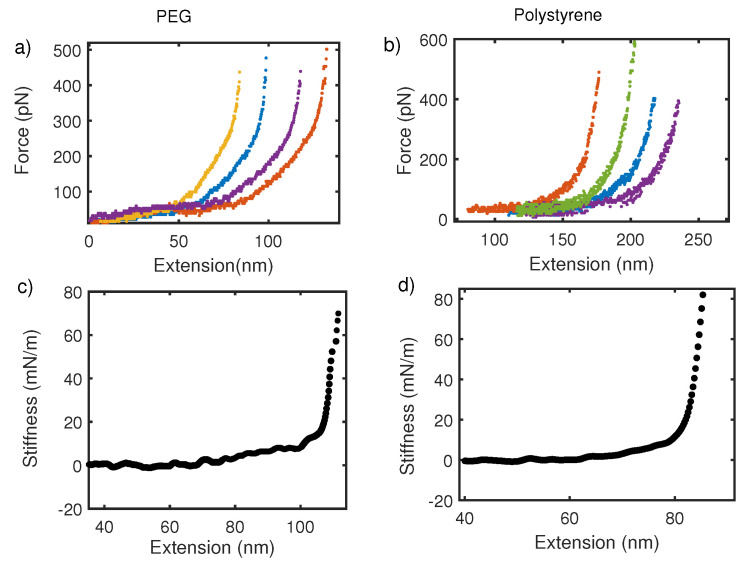
(**a**,**c**) Show force–extension and stiffness–extension curves for PEG in water. Similarly, (**b**,**d**) are force–extension and stiffness–extension curve for polystyrene in water, respectively. Fluctuations about mean stiffness are stronger in PEG with molecular weight 10 kDa than a longer polymer of polystyrene with molecular weight 200 kDa. No size dependent variation in fluctuation is observed for the force–extension curve.

So far, we have focused on the average thermodynamic behavior of the force–extension curve. However, fluctuations in the mean also reveal about deconvolution procedure mentioned above. Figure 6 shows the force–extension curve for PEG and polystyrene taken with commercial AFM in (a) and (b) respectively. From Figure 6a,b, we observe that PEG and Polystyrene with molecular weight 10 kDa and 200 kDa respectively, show no change in regards to fluctuations about the mean force–extension curve. It is, however, expected on basis of statistical mechanics of polymers that fluctuation about mean would go as $1/\sqrt{N}$ where *N* is monomer units, following Poisson statistics. Fluctuations in force for a finite-size system like polymer are expected to be large but the force–extension curve in pulling experiments shows no large fluctuation and no variation with the size of polymer. This is because overall fluctuation in the coupled cantilever–polymer system is dominated by the cantilever [58,59]. On the other hand, the stiffness–extension curve from oscillatory measurement shows a clear distinction in terms of fluctuations about the mean in two polymers (Figure 6c,d). Fluctuations in shorter-length polymers are large. This also supports the argument that an intrinsic polymer response is captured more effectively by local oscillatory response than global pulling experiments.

## 4. Conclusions

In conclusion, we performed elasticity measurement on PEG and polystyrene with a home-built fiber-interferometer-based AFM. The elastic response was measured by oscillating the cantilever base and recording the amplitude and phase response using a lock-in amplifier. The amplitude signal, in particular, was quantified to obtain stiffness–extension profiles and analyzed with the WLC entropic model. For both polymers, the stiffness–extension curve deviates significantly from the conventional force–extension curve in good solvent conditions. When modeled with the phenomenological WLC model, its fitting of the stiffness–extension curve produced a more reasonable estimate (0.55 nm for PEG and 0.8 nm for polystyrene), which is about five times more compared with conventional pulling experiments (0.1 nm for PEG and 0.2 nm for polystyrene). The value is also consistent with equilibrium measurements with magnetic tweezers and other force-free techniques. Although fluctuations about mean elastic behavior are known not to dominate the pulling force–extension curve, local oscillatory measurement of the stiffness–extension curve does reveal a clear size dependence of fluctuations. Our work highlights the importance of coupling between the AFM cantilever probe and polymer elasticity which in statistical terms is expressed as an intricate convolution. In addition, the stiffness–extension curve shows no deviation from the force–extension curve in the poor solvent case of polystyrene in water. We attribute this to the additional hydrophobic contribution that effectively lowers the persistence length and suggests that WLC entropic model is ad hoc in poor solvents.

## Data Availability

The data in the present study are available upon request from corresponding author.
